# Supplementary material for: Maleic, glycolic and acetoacetic acids-leaching for recovery of valuable metals from spent lithium-ion batteries: leaching parameters, thermodynamics and kinetics
Source: R Soc Open Sci. 2019 Sep 4;6(9):191061. doi: 10.1098/rsos.191061 (PMC6774949; doi:10.1098/rsos.191061)
Supplement: Additional data for the main body of the paper [file rsos191061supp1.docx]

Maleic, glycolic and acetoacetic acids-leaching for recovery of valuable metals from spent LIBs: Leaching parameters, thermodynamics and kinetics

Borui Liu^1^, Qing Huang^1^*, Yuefeng Su^1^*, Liuye Sun^1^, Tong Wu^1^,

Guange Wang^1^, Ryan M. Kelly^2^ and Feng Wu^1^

^1^School of Materials Science and Engineering, Beijing Institute of Technology, Beijing 100081, China.

^2^Rykell Scientific Editorial, LLC, Los Angeles, CA, USA.

**Keywords:** Spent lithium ion batteries; Metal recovery; Maleic acid; Glycolic acid; Acetoacetic acid

*Author for correspondence (Q. Huang: huangqing3121@sina.com; Y. Su: suyuefeng@bit.edu.cn).

Supplementary materials

Table S1. The element component on the surface of the particles of the active cathodic materials and leaching residue from EDS (weight %)

| Element | Active cathodic material | Leaching residue | | |
| --- | --- | --- | --- | --- |
|  |  | Maleic acid | Glycolic acid | Acetoacetic acid |
| Co | 62.63 | 72.20 | 79.10 | 77.45 |
| O | 32.68 | 27.80 | 20.90 | 22.55 |

Table S2. Experimental results of the orthogonal experiment for maleic acid

| No. | Leaching parameter | | | | |  | Leaching efficiency  % | |
| --- | --- | --- | --- | --- | --- | --- | --- | --- |
|  | Temperature  ℃ | Reaction time  min | Acid concentration  mol L^-1^ | H_2_O_2_ concentration  vol % | Solid/liquid ratio  g L^-1^ |  | Li | Co |
| 1 | 60 | 30 | 0.5 | 1 | 10 |  | 68.689 | 56.213 |
| 2 | 60 | 40 | 1.5 | 2.5 | 20 |  | 69.624 | 59.686 |
| 3 | 60 | 50 | 2 | 1.5 | 30 |  | 59.682 | 52.417 |
| 4 | 60 | 60 | 1 | 2 | 40 |  | 69.940 | 48.670 |
| 5 | 70 | 30 | 2 | 2 | 20 |  | 64.970 | 60.659 |
| 6 | 70 | 40 | 1 | 1.5 | 10 |  | 80.745 | 73.114 |
| 7 | 70 | 50 | 0.5 | 2.5 | 40 |  | 69.263 | 50.676 |
| 8 | 70 | 60 | 1.5 | 1 | 30 |  | 76.565 | 69.775 |
| 9 | 80 | 30 | 1 | 2.5 | 30 |  | 63.668 | 47.388 |
| 10 | 80 | 40 | 2 | 1 | 40 |  | 63.846 | 59.075 |
| 11 | 80 | 50 | 1.5 | 2 | 10 |  | 75.275 | 68.519 |
| 12 | 80 | 60 | 0.5 | 1.5 | 20 |  | 73.168 | 68.388 |
| 13 | 90 | 30 | 1.5 | 1.5 | 40 |  | 75.797 | 70.790 |
| 14 | 90 | 40 | 0.5 | 2 | 30 |  | 65.940 | 44.670 |
| 15 | 90 | 50 | 1 | 1 | 20 |  | 74.811 | 69.017 |
| 16 | 90 | 60 | 2 | 2.5 | 10 |  | 63.263 | 61.076 |

Table S3. Experimental results of the orthogonal experiment for glycolic acid

| No. | Leaching parameter | | | | |  | Leaching efficiency  % | |
| --- | --- | --- | --- | --- | --- | --- | --- | --- |
|  | Temperature  ℃ | Reaction time  min | Acid concentration  mol L^-1^ | H_2_O_2_ concentration  vol % | Solid/liquid ratio  g L^-1^ |  | Li | Co |
| 1 | 60 | 30 | 0.5 | 1 | 10 |  | 75.149 | 63.168 |
| 2 | 60 | 40 | 1.5 | 2.5 | 20 |  | 74.958 | 74.900 |
| 3 | 60 | 50 | 2 | 1.5 | 30 |  | 77.276 | 76.803 |
| 4 | 60 | 60 | 1 | 2 | 40 |  | 84.186 | 83.915 |
| 5 | 70 | 30 | 2 | 2 | 20 |  | 88.285 | 83.075 |
| 6 | 70 | 40 | 1 | 1.5 | 10 |  | 85.643 | 84.916 |
| 7 | 70 | 50 | 0.5 | 2.5 | 40 |  | 74.180 | 70.140 |
| 8 | 70 | 60 | 1.5 | 1 | 30 |  | 81.390 | 77.240 |
| 9 | 80 | 30 | 1 | 2.5 | 30 |  | 79.215 | 79.210 |
| 10 | 80 | 40 | 2 | 1 | 40 |  | 84.935 | 79.531 |
| 11 | 80 | 50 | 1.5 | 2 | 10 |  | 90.016 | 83.100 |
| 12 | 80 | 60 | 0.5 | 1.5 | 20 |  | 74.934 | 66.707 |
| 13 | 90 | 30 | 1.5 | 1.5 | 40 |  | 82.624 | 82.208 |
| 14 | 90 | 40 | 0.5 | 2 | 30 |  | 77.297 | 66.737 |
| 15 | 90 | 50 | 1 | 1 | 20 |  | 81.434 | 77.652 |
| 16 | 90 | 60 | 2 | 2.5 | 10 |  | 87.493 | 87.484 |

Table S4. Experimental results of the orthogonal experiment for acetoacetic acid

| No. | Leaching parameter | | | | |  | Leaching efficiency /% | |
| --- | --- | --- | --- | --- | --- | --- | --- | --- |
|  | Temperature  ℃ | Reaction time  min | Acid concentration  mol L^-1^ | H_2_O_2_ concentration  vol % | Solid/liquid ratio  g L^-1^ |  | Li | Co |
| 1 | 60 | 30 | 0.5 | 1 | 10 |  | 71.132 | 64.065 |
| 2 | 60 | 40 | 1.5 | 2.5 | 20 |  | 77.177 | 76.855 |
| 3 | 60 | 50 | 2 | 1.5 | 30 |  | 86.758 | 83.422 |
| 4 | 60 | 60 | 1 | 2 | 40 |  | 65.261 | 55.095 |
| 5 | 70 | 30 | 2 | 2 | 20 |  | 73.613 | 72.052 |
| 6 | 70 | 40 | 1 | 1.5 | 10 |  | 94.194 | 82.455 |
| 7 | 70 | 50 | 0.5 | 2.5 | 40 |  | 60.624 | 59.691 |
| 8 | 70 | 60 | 1.5 | 1 | 30 |  | 77.081 | 74.384 |
| 9 | 80 | 30 | 1 | 2.5 | 30 |  | 51.147 | 41.428 |
| 10 | 80 | 40 | 2 | 1 | 40 |  | 54.747 | 50.709 |
| 11 | 80 | 50 | 1.5 | 2 | 10 |  | 88.254 | 84.788 |
| 12 | 80 | 60 | 0.5 | 1.5 | 20 |  | 75.467 | 72.518 |
| 13 | 90 | 30 | 1.5 | 1.5 | 40 |  | 82.765 | 72.246 |
| 14 | 90 | 40 | 0.5 | 2 | 30 |  | 65.231 | 65.957 |
| 15 | 90 | 50 | 1 | 1 | 20 |  | 56.701 | 43.103 |
| 16 | 90 | 60 | 2 | 2.5 | 10 |  | 86.647 | 83.880 |

Table S5. Analysis results of the orthogonal experiment for the three organic acids

|  | Parameter factor | Temperature | Reaction time | Acid concentration | H_2_O_2_ concentration | Solid/liquid ratio |
| --- | --- | --- | --- | --- | --- | --- |
| Maleic acid | | | | | | |
| Li | K1 | 66.984 | 68.281 | 69.265 | 70.978 | 71.993 |
|  | K2 | 72.886 | 70.039 | 72.291 | 72.348 | 70.643 |
|  | K3 | 68.989 | 69.758 | 74.315 | 69.031 | 66.464 |
|  | K4 | 69.953 | 70.734 | 62.940 | 66.455 | 69.712 |
|  | Extreme deviation | 5.902 | 2.453 | 11.375 | 5.893 | 5.529 |
| Co | K1 | 54.247 | 58.763 | 54.987 | 63.520 | 64.730 |
|  | K2 | 63.556 | 59.136 | 59.547 | 66.177 | 64.438 |
|  | K3 | 60.842 | 60.157 | 67.193 | 55.629 | 53.562 |
|  | K4 | 61.388 | 61.977 | 58.307 | 54.707 | 57.303 |
|  | Extreme deviation | 9.309 | 3.215 | 12.206 | 11.471 | 11.168 |
| Glycolic acid | | | | | | |
| Li | K1 | 77.89235 | 81.31822 | 75.39016 | 80.72697406 | 84.57506 |
|  | K2 | 82.37433 | 80.70829 | 82.61938 | 80.11919222 | 79.90301 |
|  | K3 | 82.2751 | 80.72651 | 82.24711 | 84.94615301 | 78.79461 |
|  | K4 | 82.21196 | 82.00072 | 84.4971 | 78.96142333 | 81.48107 |
|  | Extreme deviation | 4.481983 | 1.292434 | 9.106935 | 5.984729681 | 5.78045 |
| Co | K1 | 74.69673 | 76.91524 | 66.6879 | 74.39781676 | 79.667 |
|  | K2 | 78.84267 | 76.52117 | 81.42349 | 77.65869892 | 75.58349 |
|  | K3 | 77.13708 | 76.92387 | 79.36218 | 79.20677962 | 74.99761 |
|  | K4 | 78.52023 | 78.83643 | 81.72314 | 77.9334239 | 78.94861 |
|  | Extreme deviation | 4.145941 | 2.315257 | 15.03524 | 4.808962855 | 4.669386 |
| Acetoacetic acid | | | | | | |
| Li | K1 | 75.082 | 74.810 | 68.113 | 64.915 | 79.912 |
|  | K2 | 76.378 | 67.692 | 61.681 | 79.651 | 75.885 |
|  | K3 | 67.404 | 73.084 | 81.319 | 78.235 | 70.054 |
|  | K4 | 72.836 | 76.114 | 80.587 | 68.899 | 65.849 |
|  | Extreme deviation | 8.974 | 8.422 | 19.638 | 14.735 | 14.063 |
| Co | K1 | 69.860 | 62.448 | 65.558 | 58.065 | 78.797 |
|  | K2 | 72.145 | 68.994 | 55.520 | 77.660 | 66.132 |
|  | K3 | 62.361 | 67.751 | 77.068 | 69.473 | 66.298 |
|  | K4 | 66.296 | 71.469 | 72.516 | 65.463 | 59.435 |
|  | Extreme deviation | 9.785 | 9.022 | 21.548 | 19.595 | 19.362 |

Table S6. Formation energy of the possible leaching products

| Organic acid | Molecular formula of the products | Formation energy / 10^-19^ J |
| --- | --- | --- |
| Maleic acid | (C_4_H_3_O_4_)_2_Co * | 2.214 |
|  | C_4_H_2_O_4_Co ** | 5.239 |
|  | C_4_H_3_O_4_Li | 11.43 |
| Glycolic acid | (C_2_H_3_O_3_)_2_Co | 2.067 |
|  | C_2_H_3_O_3_Li | 12.02 |
| Acetoacetic acid | (C_4_H_5_O_4_)_2_Co | 45.43 |
|  | C_4_H_5_O_4_Li | 21.44 |

* the maleic acid-leaching product **(a)** in Fig 7

** the maleic acid-leaching product **(b)** in Fig 7

Table S7. The equilibrium constants and Gibbs free energy of the acid-leaching reactions

| Maleic acid | |  | Glycolic acid | |  | Acetoacetic acid | |
| --- | --- | --- | --- | --- | --- | --- | --- |
| K | ΔG / kJ mol^-1^ |  | K | ΔG / kJ mol^-1^ |  | K | ΔG / kJ mol^-1^ |
| 1.45 $\times$ 10^-3^ | 18.65 |  | 5.19 $\times$ 10^-6^ | 34.72 |  | 5.72 $\times$ 10^-5^ | 27.87 |

Table S8. The fitting parameters of the surface chemical reaction model for the acid-leaching process by the three organic acids

| Acid-leaching of Li | | | | | | | | |
| --- | --- | --- | --- | --- | --- | --- | --- | --- |
| Temperature  / ºC | Maleic acid | |  | Glycolic acid | |  | Acetoacetic acid | |
|  | k / min^-1^ | R^2^ |  | k / min^-1^ | R^2^ |  | k / min^-1^ | R^2^ |
| 50 | 0.00200 | 0.993 |  | 0.00198 | 0.994 |  | 0.00208 | 0.996 |
| 60 | 0.00299 | 0.997 |  | 0.00308 | 0.994 |  | 0.00299 | 0.992 |
| 70 | 0.00429 | 0.993 |  | 0.00460 | 0.992 |  | 0.00450 | 0.997 |
| 80 | 0.00602 | 0.998 |  | 0.00639 | 0.997 |  | 0.00638 | 0.998 |
| 90 | 0.00797 | 0.992 |  | 0.00905 | 0.995 |  | 0.00855 | 0.996 |
| Acid-leaching of Co | | | | | | | | |
| Temperature  / ºC | Maleic acid | |  | Glycolic acid | |  | Acetoacetic acid | |
|  | k / min^-1^ | R^2^ |  | k / min^-1^ | R^2^ |  | k / min^-1^ | R^2^ |
| 50 | 0.00210 | 0.993 |  | 0.00199 | 0.992 |  | 0.00208 | 0.993 |
| 60 | 0.00300 | 0.995 |  | 0.00303 | 0.998 |  | 0.00319 | 0.993 |
| 70 | 0.00430 | 0.991 |  | 0.00450 | 0.995 |  | 0.00460 | 0.996 |
| 80 | 0.00598 | 0.997 |  | 0.00621 | 0.995 |  | 0.00663 | 0.994 |
| 90 | 0.00781 | 0.994 |  | 0.00906 | 0.996 |  | 0.00876 | 0.998 |

Table S9. The fitting parameters of the liquid film diffusion model for the acid-leaching process by the three organic acids

| Acid-leaching of Li | | | | | | | | |
| --- | --- | --- | --- | --- | --- | --- | --- | --- |
| Temperature  ºC | Maleic acid | |  | Glycolic acid | |  | Acetoacetic acid | |
|  | k / min^-1^ | R^2^ |  | k / min^-1^ | R^2^ |  | k / min^-1^ | R^2^ |
| 50 | 0.00462 | 0.988 |  | 0.00303 | 0.989 |  | 0.00245 | 0.986 |
| 60 | 0.00593 | 0.987 |  | 0.00374 | 0.980 |  | 0.00268 | 0.982 |
| 70 | 0.00664 | 0.985 |  | 0.00500 | 0.978 |  | 0.00346 | 0.978 |
| 80 | 0.00577 | 0.984 |  | 0.00588 | 0.966 |  | 0.00389 | 0.962 |
| 90 | 0.00467 | 0.982 |  | 0.00639 | 0.936 |  | 0.00422 | 0.939 |
| Acid-leaching of Co | | | | | | | | |
| Temperature  / ºC | Maleic acid | |  | Glycolic acid | |  | Acetoacetic acid | |
|  | k / min^-1^ | R^2^ |  | k / min^-1^ | R^2^ |  | k / min^-1^ | R^2^ |
| 50 | 0.00514 | 0.987 |  | 0.00350 | 0.988 |  | 0.00261 | 0.990 |
| 60 | 0.00614 | 0.986 |  | 0.00444 | 0.976 |  | 0.00316 | 0.989 |
| 70 | 0.00700 | 0.984 |  | 0.00591 | 0.982 |  | 0.00386 | 0.986 |
| 80 | 0.00670 | 0.986 |  | 0.00708 | 0.967 |  | 0.00407 | 0.972 |
| 90 | 0.00545 | 0.983 |  | 0.00801 | 0.951 |  | 0.00463 | 0.968 |

Table S10. The fitting parameters of the ash or product layer diffusion model for the acid-leaching process by the three organic acids

| Acid-leaching of Li | | | | | | | | |
| --- | --- | --- | --- | --- | --- | --- | --- | --- |
| Temperature  / ºC | Maleic acid | |  | Glycolic acid | |  | Acetoacetic acid | |
|  | k / min^-1^ | R^2^ |  | k / min^-1^ | R^2^ |  | k / min^-1^ | R^2^ |
| 50 | 0.00018 | 0.986 |  | 0.00241 | 0.988 |  | 0.00292 | 0.990 |
| 60 | 0.00043 | 0.986 |  | 0.00423 | 0.987 |  | 0.00441 | 0.988 |
| 70 | 0.00102 | 0.983 |  | 0.00656 | 0.987 |  | 0.00669 | 0.985 |
| 80 | 0.00259 | 0.976 |  | 0.00932 | 0.982 |  | 0.00928 | 0.985 |
| 90 | 0.00531 | 0.962 |  | 0.01305 | 0.979 |  | 0.01184 | 0.982 |
| Acid-leaching of Co | | | | | | | | |
| Temperature  / ºC | Maleic acid | |  | Glycolic acid | |  | Acetoacetic acid | |
|  | k / min^-1^ | R^2^ |  | k / min^-1^ | R^2^ |  | k / min^-1^ | R^2^ |
| 50 | 0.00015 | 0.988 |  | 0.00213 | 0.990 |  | 0.00284 | 0.989 |
| 60 | 0.00040 | 0.987 |  | 0.00379 | 0.988 |  | 0.00465 | 0.990 |
| 70 | 0.00094 | 0.987 |  | 0.00599 | 0.983 |  | 0.00681 | 0.988 |
| 80 | 0.00220 | 0.979 |  | 0.00867 | 0.983 |  | 0.00962 | 0.986 |
| 90 | 0.00454 | 0.963 |  | 0.01302 | 0.978 |  | 0.01225 | 0.983 |





Fig S1. TG-DSC curves of the spent cathodic material after reaction with the NaOH solution


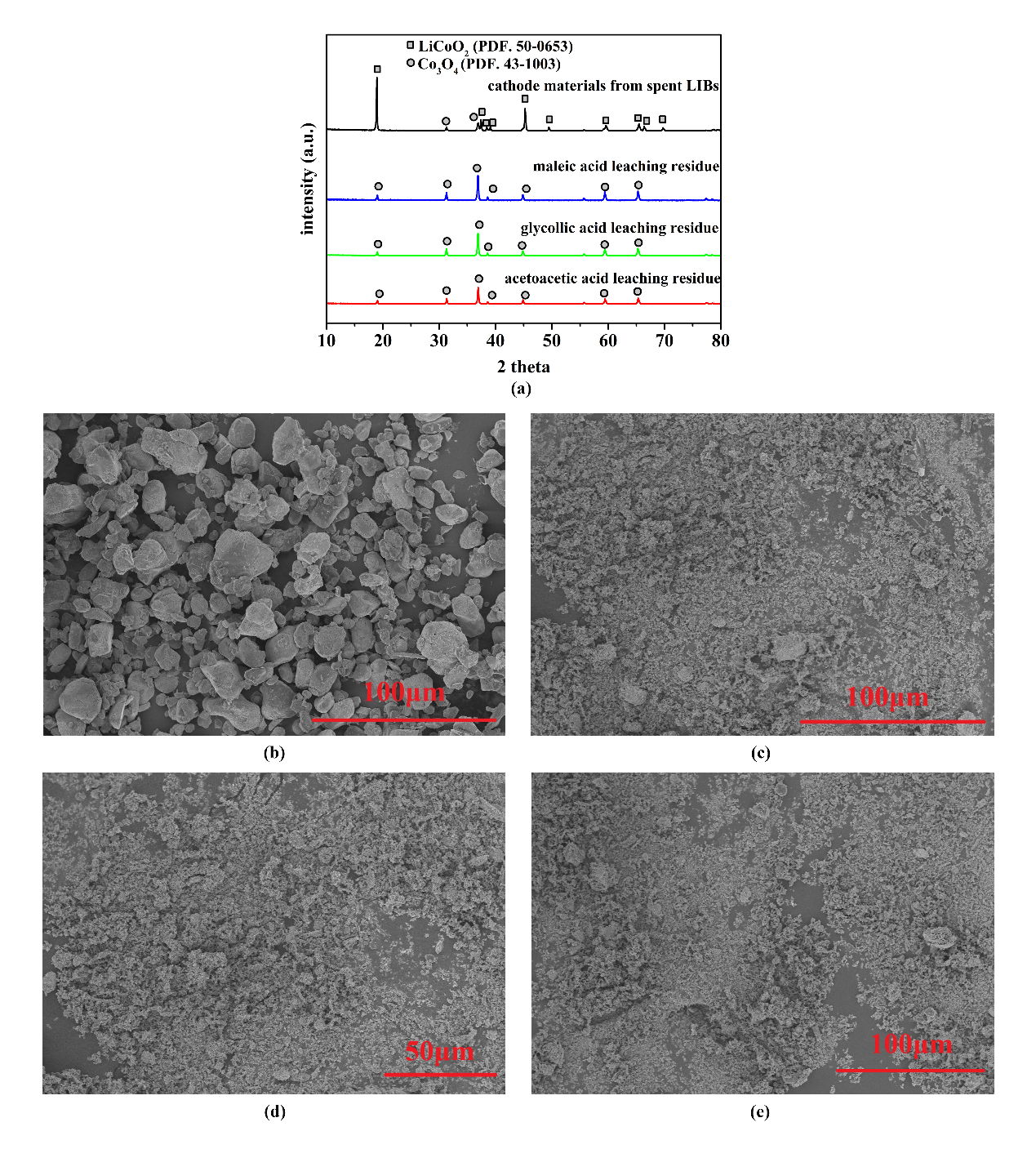


Fig S2. XRD patterns of the active cathodic material before leaching and the leaching residue **(a)**; SEM images of the active cathodic material **(b)** and the leaching residue from maleic acid **(c)**, glycolic acid **(d)** and acetoacetic acid **(e)**


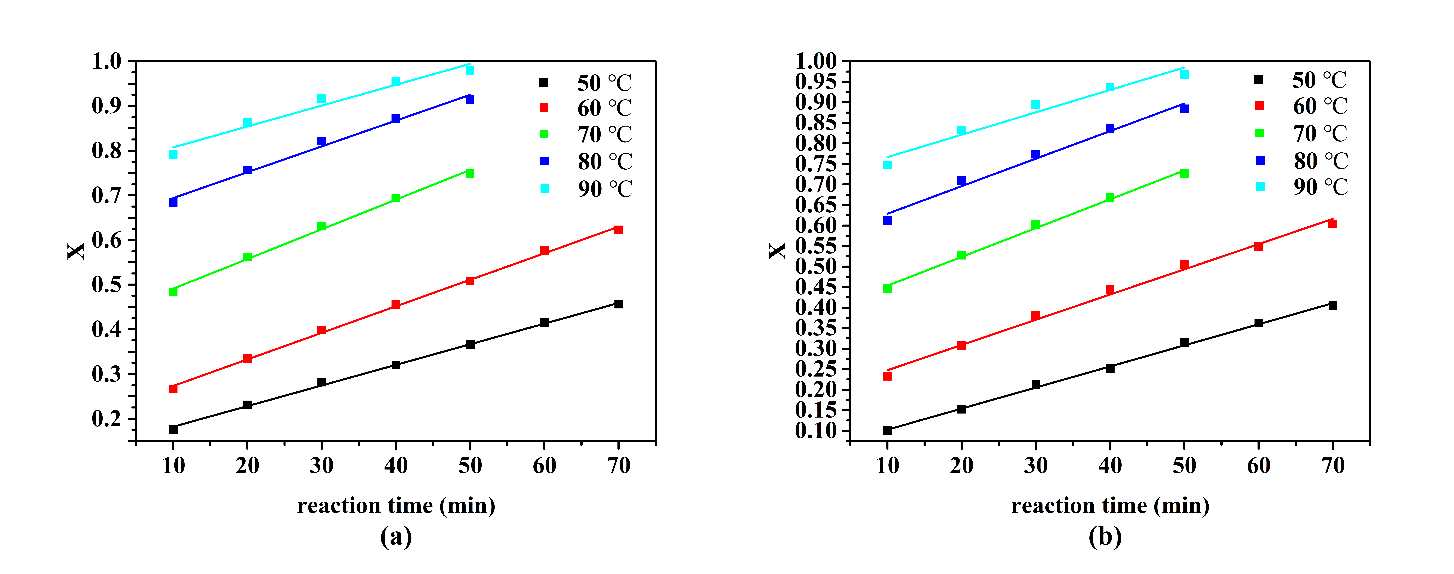


Fig S3. Kinetics study of Li **(a)** and Co **(c)** in maleic acid-leaching process (liquid film diffusion model)


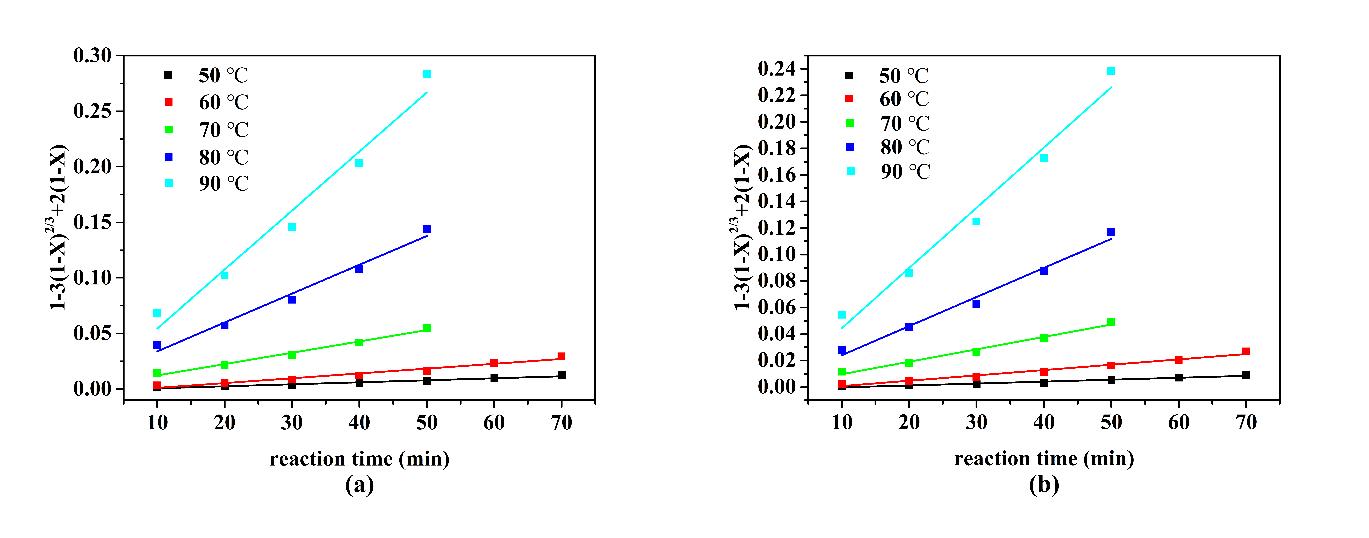


Fig S4. Kinetics study of Li **(a)** and Co **(c)** in maleic acid-leaching process (ash or product layer diffusion model)


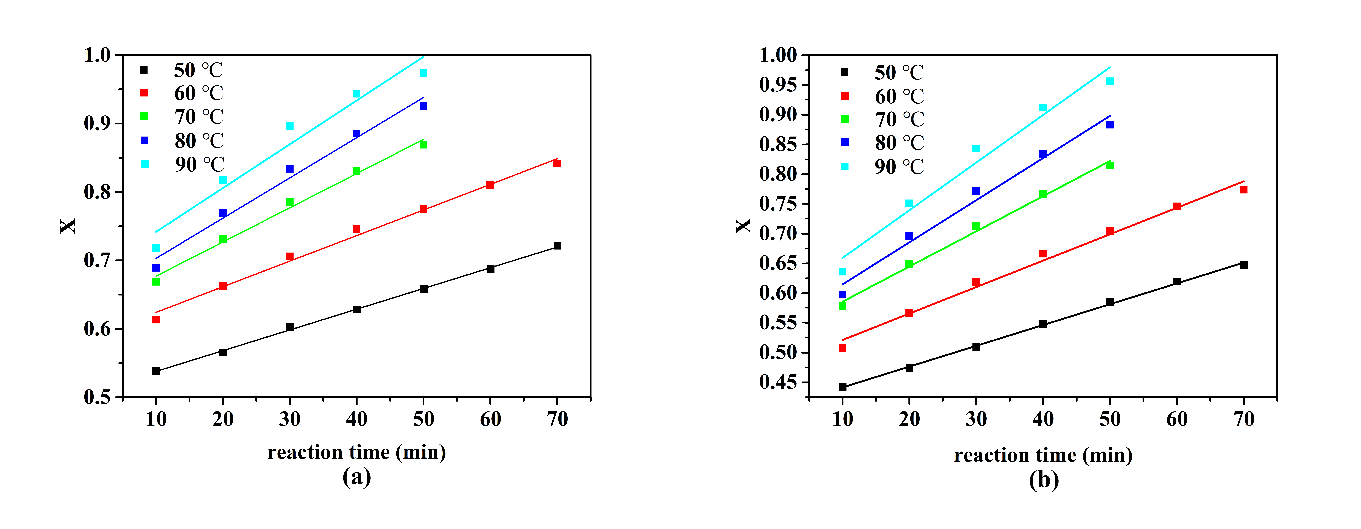


Fig S5. Kinetics study of Li **(a)** and Co **(c)** in glycolic acid-leaching process (liquid film diffusion model)


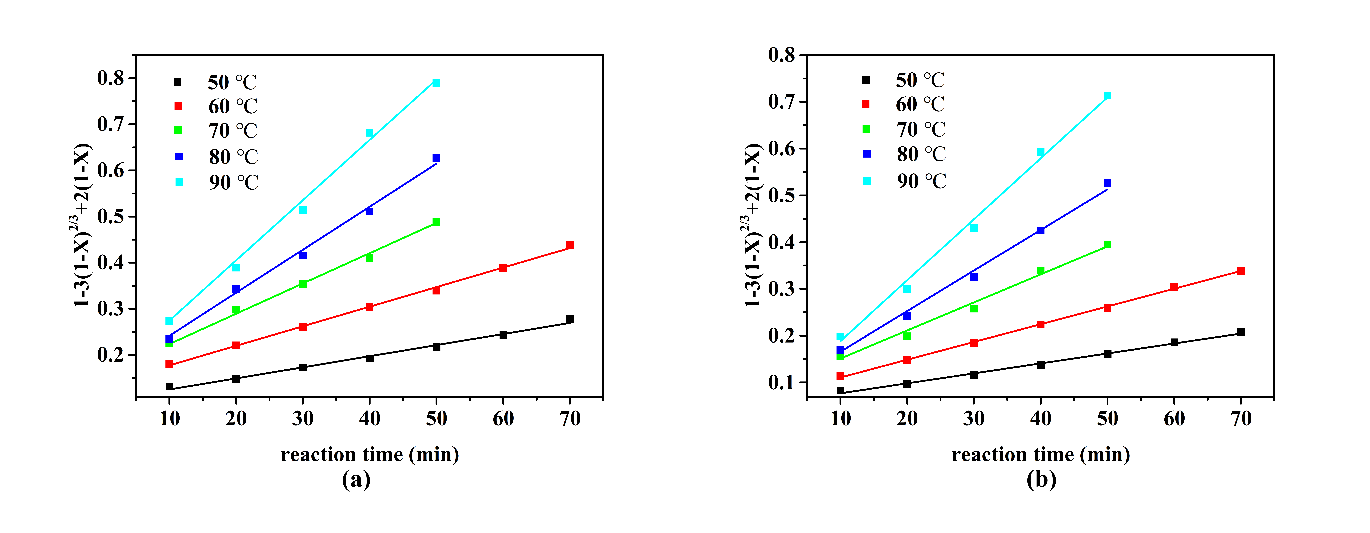


Fig S6. Kinetics study of Li **(a)** and Co **(c)** in glycolic acid-leaching process (ash or product layer diffusion model)


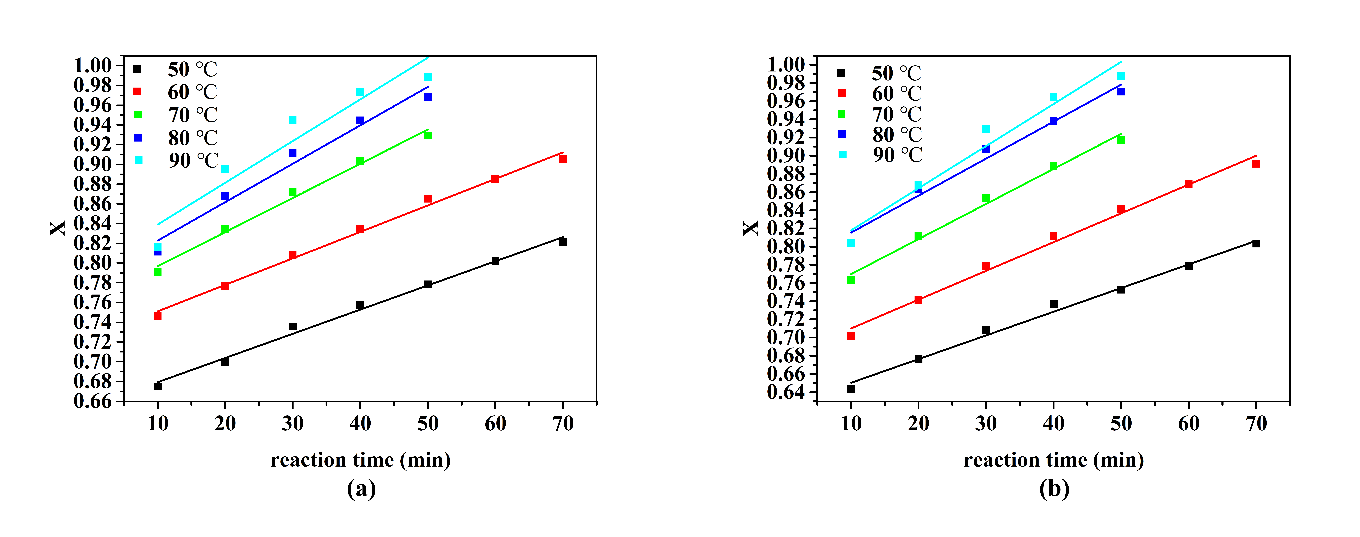


Fig S7. Kinetics study of Li **(a)** and Co **(c)** in acetoacetic acid-leaching process (liquid film diffusion model)


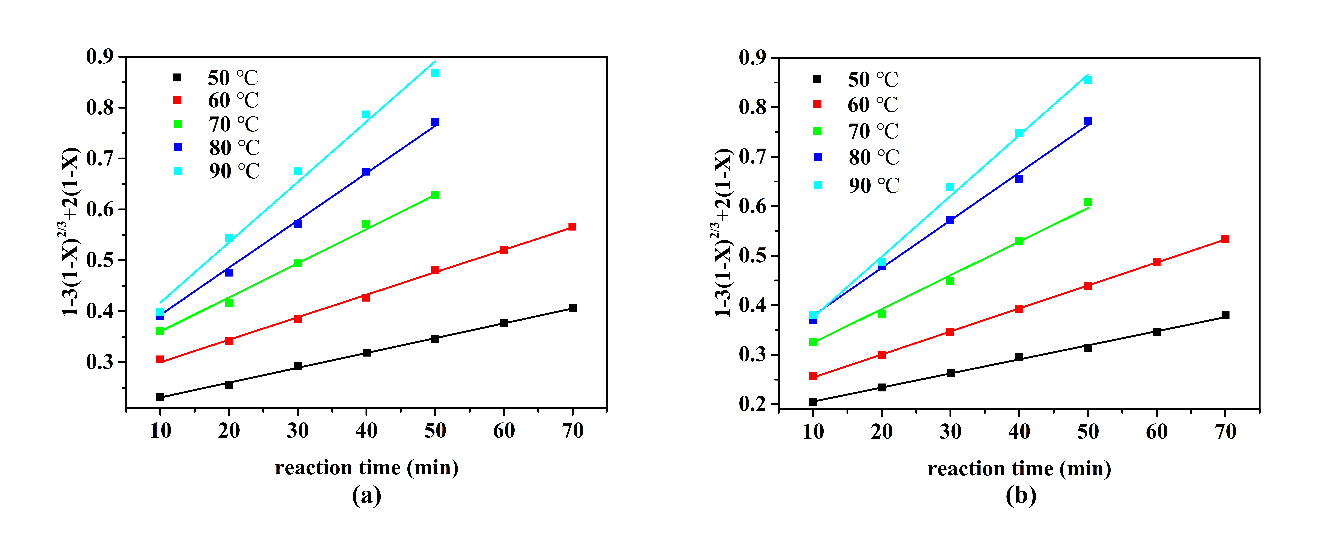


Fig S8. Kinetics study of Li **(a)** and Co **(c)** in acetoacetic acid-leaching process (ash or product diffusion model)
